# Differential Modulation of NF-*κ*B in Neurons and Astrocytes Underlies Neuroprotection and Antigliosis Activity of Natural Antioxidant Molecules

**DOI:** 10.1155/2019/8056904

**Published:** 2019-08-14

**Authors:** Francesca Martorana, Maria Foti, Assunta Virtuoso, Daniela Gaglio, Federica Aprea, Tiziana Latronico, Rocco Rossano, Paolo Riccio, Michele Papa, Lilia Alberghina, Anna Maria Colangelo

**Affiliations:** ^1^Laboratory of Neuroscience “R. Levi-Montalcini,” Department of Biotechnology and Bioscience, University of Milano-Bicocca, Milano, Italy; ^2^SYSBIO Centre of Systems Biology ISBE.ITALY, University of Milano-Bicocca, Milano, Italy; ^3^School of Medicine, University of Milano-Bicocca, Monza, Italy; ^4^Laboratory of Morphology of Neuronal Network, Department of Public Medicine, University of Campania “Luigi Vanvitelli”, Napoli, Italy; ^5^Department of Biosciences, Biotechnologies and Biopharmaceutics, University of Bari, Italy; ^6^Department of Sciences, University of Basilicata, Potenza, Italy; ^7^NeuroMI, Milan Center for Neuroscience, University of Milano-Bicocca, Milano, Italy

## Abstract

Neuroinflammation, a hallmark of chronic neurodegenerative disorders, is characterized by sustained glial activation and the generation of an inflammatory loop, through the release of cytokines and other neurotoxic mediators that cause oxidative stress and limit functional repair of brain parenchyma. Dietary antioxidants may protect against neurodegenerative diseases by counteracting chronic neuroinflammation and reducing oxidative stress. Here, we describe the effects of a number of natural antioxidants (polyphenols, carotenoids, and thiolic molecules) in rescuing astrocytic function and neuronal viability following glial activation by reducing astrocyte proliferation and restoring astrocytic and neuronal survival and basal levels of reactive oxygen species (ROS). All antioxidant molecules are also effective under conditions of oxidative stress and glutamate toxicity, two maladaptive components of neuroinflammatory processes. Moreover, it is remarkable that their antioxidant and anti-inflammatory activity occurs through differential modulation of NF-*κ*B binding activity in neurons and astrocytes. In fact, we show that inflammatory stimuli promote a significant induction of NF-*κ*B binding activity in astrocytes and its concomitant reduction in neurons. These changes are prevented in astrocytes and neurons pretreated with the antioxidant molecules, suggesting that NF-*κ*B plays a key role in the modulation of survival and anti-inflammatory responses. Finally, we newly demonstrate that effective antigliosis and neuroprotective activity is achieved with a defined cocktail of four natural antioxidants at very low concentrations, suggesting a promising strategy to reduce inflammatory and oxidative damage in neurodegenerative diseases with limited side effects.

## 1. Introduction

Neuroinflammation and increased oxidative stress are common hallmarks of chronic neurodegenerative disorders including Alzheimer's (AD) and Parkinson's (PD) diseases, Amyotrophic Lateral Sclerosis (ALS), and Multiple Sclerosis (MS) [1–6].

Neuroinflammatory processes involve the activation of glial cells (astrocytes and microglia) and the release of growth factors and inflammatory mediators (such as cytokines) aiming at counteracting the toxic events and promoting neuronal repair. Nevertheless, chronic astrocytic activation (reactive gliosis) may hold deleterious consequences that limit functional repair of brain parenchyma [4, 6, 7]. Reactive gliosis is characterized by proliferation and loss of proper astrocytic function, including a decrease of glial (GLAST/GLT1) and vesicular (vGLUT) glutamate transporters, which compromises synaptic function and leads to excitotoxicity [8–10]. Moreover, activated microglia produce reactive oxygen species (ROS) which further increase brain oxidative stress [5, 11, 12].

Compelling evidence has greatly enhanced the interest for the role of some dietary molecules in the prevention of many diseases, including neurodegenerative and neuroinflammatory disorders. Most dietary supplements (i.e., polyphenols, carotenoids, and thiolic compounds) are potent antioxidants. Their antioxidant and anti-inflammatory activities have been reported in cellular and animal models of neurodegeneration involving oxidative stress, such as A*β* toxicity models of AD, neurotoxin (6-OHDA or MPTP) models of PD, MS, traumatic brain injury, and ischemia [13–16]. Moreover, several reports have shown that antioxidants activate pathways and transcription factors (such as NF-*κ*B, Nrf2/Keap1/ARE, and PPAR/PGC-1*α*) that regulate metabolism and inflammatory responses [13–16].

Among transcription factors, NF-*κ*B is induced in response to several stimuli in neurons and astrocytes. In neurons, NF-*κ*B is activated by stress stimuli and regulates the transcription of survival genes, including growth factors, such as Nerve Growth Factor (Colangelo AM, unpublished), Bcl-2, IAP, and Mn-SOD [17]. In astrocytes, NF-*κ*B participates in complex inflammatory loops regulating production and release of proinflammatory cytokines, such as Interleukin-1*β*, Tumor Necrosis Factor *α* (TNF*α*), and inducible NO synthase (iNOS) [17–19].

Natural antioxidants include polyphenols (flavonoids and nonflavonoids), as well as carotenoids and thiolic compounds. The main flavonoid molecules include quercetin (QRC) and catechins (green tea extract (GTE)), while key nonflavonoid molecules are resveratrol (RSV), curcumin (CRC), and hydroxytyrosol (Oliplus (OLP)) [20, 21].

Polyphenols are known for their effects against microbial agents, as well as for counteracting the effect of diets rich in saturated and *trans*-fatty acids by downregulating production of molecules related to inflammation, oxidative stress, and angiogenesis. Their known neuroprotective activity [22–27] has been reported to be dose-dependent [28], due to their hormesis effects at high concentrations [28, 29].

Other natural antioxidants are carotenoids (such as lycopene (LYC)) [30, 31] and thiolic compounds including *α*-lipoic acid (ALA), glutathione, and N-acetyl cysteine (NAC), known for their anti-inflammatory activity [32, 33]. For instance, ALA has been proved to act as an effective immunomodulator in the Experimental Autoimmune Encephalomyelitis (EAE) model of MS [34].

Because of their metabolic effects and their low bioavailability, the intake of polyphenols is recommended to occur as a mixture of different flavonoids and nonflavonoids [35, 36]. Combinations of polyphenols and other antioxidant compounds at low doses may increase bioavailability of dietary molecules and avoid their potential toxicity, while providing neuroprotection against oxidative stress and inflammatory processes, thus representing a promising approach in inflammation-based neurological disorders.

Here, we used primary cultures of neurons and astrocytes to assess the antigliosis and neuroprotective properties of several natural antioxidants. Our data revealed that all tested antioxidants (i) decrease gliosis by reducing astrocytic proliferation and (ii) protect cortical neurons exposed to conditioned medium (CM) from reactive astrocytes, as well as under conditions of glutamate oxidative stress toxicity; (iii) all antioxidants act through differential modulation of NF-*κ*B in neurons and astrocytes. Finally, (iv) we newly demonstrate that effective antigliosis and neuroprotective activity can be achieved by defined cocktails of dietary antioxidants at low doses, as a new strategy to reduce inflammatory and oxidative damage in neurodegenerative disorders with limited side effects.

## 2. Materials and Methods

### 2.1. Drugs and Reagents

Resveratrol (RSV), quercetin (QRC), curcumin (CRC), lycopene (LYC), alpha-lipoic acid (ALA), Oliplus (OLP, a mixture including hydroxytyrosol), green tea extract (GTE), and N-acetyl cysteine (NAC) were from Nutraceutica srl (Monterenzio, Bologna, Italy). 2′,7′-Dichlorodihydrofluorescein diacetate was purchased from Thermo Fisher Scientific. The TranSignal Protein/DNA Array I and EMSA Gel Shift Kit were purchased by Panomics Inc. (Fremont, CA, USA).

### 2.2. Primary Cortical Neurons and Astrocyte Cultures

Cortical neurons were prepared from neonatal (P1-P2) C57BL/6 mice (Harlan Laboratories, Italy) following a previously described protocol [37]. In brief, cortices were dissected, washed in dissociation medium, and digested by trypsin (0.15%) in the presence of deoxyribonuclease (DNase, 1 mg/ml; Sigma). Following mechanical dissociation, cells (1 × 10^6^/ml) were plated onto poly-D-lysine- (1 mg/ml) coated dishes. Neurons were cultured in Neurobasal medium (Thermo Fisher Scientific) supplemented with B27 (Thermo Fisher Scientific), bFGF 10 ng/ml (Thermo Fisher Scientific), glutamine, and antibiotics (Euroclone) at 37°C in 5% CO_2_. Cultures were used after 8 days in vitro (DIV).

Primary astrocytes were prepared according to a previously described protocol [10]. Cortices were dissected in Hank's Balanced Salt Solution containing HEPES/Na pH 7.4 (10 mM) and dissociated in trypsin (2.5 mg/ml) and DNase (1 mg/ml). Astrocytes were cultured in 75cm^2^ flasks in Dulbecco's modified Eagle's medium (DMEM, Euroclone) containing 10% fetal bovine serum (FBS) and antibiotics. Flasks were then shaken at 200 rpm to remove type 2 astrocytes, oligodendrocytes, and microglia. For experiments, cells were plated onto poly-D-lysine-coated dishes and exposed to Tumor Necrosis Factor *α* (TNF*α*) (10 ng/ml) or lipopolysaccharide (LPS, 1 *μ*g/ml).

### 2.3. Astrocyte Proliferation

Cortical astrocytes were plated onto poly-D-lysine-coated 35 mm dishes (5000 cells/well). Cells were synchronized in serum-free medium for 24 h, followed by incubation in growth media containing TNF*α* (10 ng/ml) or LPS (1 *μ*g/ml) in the presence/absence of the indicated antioxidant molecules at the following concentrations: RSV 10 *μ*M, QRC 10 *μ*M, CRC 10 *μ*M, LYC 10 *μ*M, ALA 10 *μ*M, OLP 100 *μ*g/ml, GTE 12.5 *μ*g/ml, and NAC 300 *μ*M. Cells were detached by trypsin (0.25%) at defined time points (1, 2, 3, 4, 5, 6, 7, 9, 12, and 14 days). Viable cells were counted by trypan blue exclusion and expressed as percent of control.

### 2.4. BrdU-ELISA Cell Proliferation Assay

BrdU incorporation was assessed as described in [10] by using the BrdU Cell Proliferation Assay (Chemicon). Cells were plated onto poly-D-lysine-coated 96-multiwell plates (2000 cells/well). After synchronization in serum-free medium, cells were incubated in growth media containing TNF*α* (10 ng/ml) or LPS (1 *μ*g/ml) in the presence/absence of the indicated antioxidant molecules. Proliferating cells were labeled by adding BrdU (10 *μ*M) to the wells during the last 24 h of treatments. Plates were then processed according to the manufacturer's instructions. BrdU incorporation was measured by using a microplate reader (Bio-Rad) at 450 nm and expressed as percent of control.

### 2.5. Cell Viability

Cell viability was assessed by using the MTT assay as described in [38]. Cortical neurons or astrocytes (5000 cells/well) were cultured on poly-D-lysine-coated 96-multiwell plates. Tetrazolium salts (0.5 mg/ml) were added to the culture medium during the last 4 h of treatments, followed by addition of MTT solubilization buffer (100 *μ*l) for 1 h. Absorbance was measured by using a microplate reader at 570 nm (700 nm reference wavelength). MTT conversion levels were reported as a percent of control.

### 2.6. Determination of ROS

ROS production was measured according to a previously described protocol [38] by incubating cells with the ROS-sensitive fluorescent probe 2′,7′-dichlorodihydrofluorescein diacetate (DCFH2-DA, Thermo Fisher Scientific). Cells (10^5^/well) were grown onto poly-D-lysine-coated 6-multiwell plates and loaded with DCFH2-DA (10 *μ*M) for 30 min before the end of treatments. Cells were immediately washed with PBS and collected in 0.25% trypsin. Fluorescence measurements were performed by FACS (FACScan, Becton-Dickinson) using the Cell Quest software (BD Bioscience). Geo-mean values of 10000 cells in the gated regions were used for data analysis by WinMDI software and expressed as percent of control.

### 2.7. Quantitative RT-PCR

For quantitative RT-PCR, cells (1 × 10^6^/well) were treated with LPS (1 *μ*g/ml) and the indicated antioxidants for 3-6 h. Total RNA extraction was performed in a TRIzol Reagent (Invitrogen), followed by purification on a Qiagen RNeasy column (Mini kit, Qiagen) and DNase digestion by RNase-free DNase Set, Qiagen. Total RNA was quantified by using a NanoDrop ND-1000 Spectrophotometer, Thermo Scientific. Reverse transcription was performed on 1 *μ*g of total RNA by using random primers and the High-Capacity cDNA Reverse Transcription Kit (Applied Biosystems). Quantitative RT-PCR (qRT-PCR) was carried out on 10 ng of total cDNA using primer sets for selected genes and the Power SYBR Green PCR Master Mix (Applied Biosystems) on a 7500 fast real-time PCR (Applied Biosystems). All samples were assessed in duplicate. Raw data (Ct (threshold cycle)) obtained from Applied Biosystems software were used to calculate the relative mRNA levels (GAPDH as housekeeping gene) by the 2−*ΔΔ*Ct method (ΔCt = Ct_target_ − GAPDH, ΔΔCt = ΔCt_stimulated_ − ΔCt_not treated_).

### 2.8. Protein/DNA Arrays

Transcription factors were identified by using the TranSignal Protein/DNA Array I (Panomics Inc.), according to manufacturer instructions. Briefly, neurons (4 × 10^6^) were plated in 60 mm dishes. After treatments, nuclear extracts were prepared as previously described [39]. Nuclear proteins (15 *μ*g) were incubated with the TranSignal Probe Mix containing biotin-labeled DNA binding oligonucleotides. Protein/DNA complexes were separated from free probes using spin columns (Panomics Inc.) and hybridized to an array membrane spotted with the consensus-binding sequences of 56 different transcription factors, followed by reaction with streptavidin-HRP conjugate. Signals were detected by chemiluminescence reaction and exposure to X-ray film. Bands were quantified by densitometry using NIH-ImageJ software.

### 2.9. Electrophoretic Mobility Shift Assay (EMSA)

Binding of NF-*κ*B in nuclear extracts was assessed by the electrophoretic mobility shift assay (EMSA) using biotin-labeled double-stranded NF-*κ*B (5′-CCAGTGGAATTCCCCAG-3′) oligonucleotides (EMSA Gel Shift Kit, Panomics Inc.). Binding reactions were carried out for 30 min at 15°C in a 25 *μ*l mixture containing 6 *μ*g of nuclear protein, 10 mM Tris, 50 mM KCl, 1 mM dithiothreitol, 5 mM MgCl_2_, 0.06% bromophenol blue, 0.25 *μ*g of BSA, 2 *μ*g poly(dI-dC), and 2 pmol of oligonucleotide probe (10 ng biotin-labeled NF-*κ*B(p65) probe). Binding specificity was confirmed by competition with a 200-fold excess of unlabeled NF-*κ*B oligonucleotides. Nuclear extracts from HeLa cells were also used as a positive CTR (EMSA Gel Shift Kit, Panomics). Protein-DNA complexes were separated through 6% nondenaturing polyacrylamide gel electrophoresis (PAGE, 120 V in 0.5% Tris-borate-EDTA), transferred to positively charged nylon membranes (Pall Biodyne B® membrane, Pall Corporation, East Hills, NY) at 300 mA for 30 min, and UV cross-linked for 3 min. After blocking, bands were visualized by streptavidin-horseradish peroxidase (HRP) reaction, followed by the enhanced chemiluminescence detection system (ECL, Amersham) and exposure to Kodak X-OMAT Autoradiography Film. Bands were quantified by densitometry using NIH-ImageJ software.

### 2.10. Western Blot Analysis

Total protein extraction and Western blotting were performed following a previously described protocol [10]. After treatments, cells were immediately washed and scraped in ice-cold PBS and lysed in lysis buffer (20 mM Tris pH 8.0, 137 mM NaCl, 1% Nonidet-P40, 10% glycerol, and 1 mM DTT) containing Protease and Phosphatase Inhibitor Cocktail (PhosSTOP, Roche). Following 20 min incubation on ice, cellular debris were pelleted by centrifugation at 14000 g for 10 min at 4°C. Protein concentration was determined by the Bio-Rad protein assay (Bio-Rad).

Cell lysates (20-25 *μ*g total protein) in loading buffer (50 mM Tris pH 6.8, 2% SDS, 100 mM DTT, 10% glycerol, and 0.1% bromophenol blue) were separated on 10% SDS-PAGE gels and transferred to nitrocellulose (Schleicher & Schuell). After blocking with 5% nonfat milk in TBST buffer (10 mM Tris pH 7.5, 150 mM NaCl, and 0.2% Tween-20), blots were probed overnight at 4°C with the mouse vGLUT antibody (1 : 5000; Synaptic System) in TBST, followed by exposure to HRP-conjugated donkey anti-mouse IgG (1 : 10000; Amersham Biotech). Protein bands were detected by enhanced chemiluminescence (ECL, Amersham Biosciences) and quantified by densitometry using NIH-ImageJ software. *β*-Actin was used to normalize for differences between samples.

### 2.11. Statistical Analysis

Data are shown as the mean ± SEM. Statistical analysis was carried out by using GraphPad Prism for Windows 6.0 (GraphPad Software, San Diego, CA, USA). Intergroup variance was determined by ANOVA and Dunnett's multiple comparison test. Values of *p* < 0.05, <0.01, or <0.001 were taken as statistically significant.

## 3. Results

### 3.1. Effect of Antioxidant Molecules on Astrocyte Proliferation

**Figure 1:**
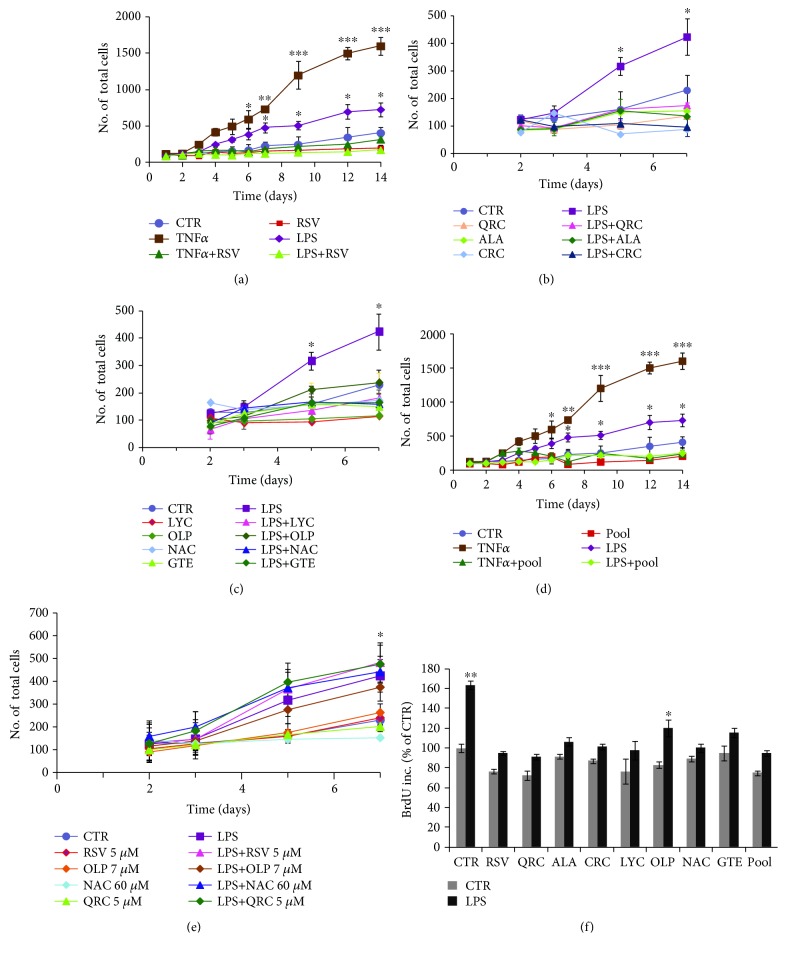
Reduction of astrocyte proliferation by antioxidant molecules. (a) Cell counts by trypan blue exclusion in astrocytes treated with TNF*α* (10 ng/ml) or LPS (1 *μ*g/ml) and the effect of cotreatment with RSV (10 *μ*M). (b, c) Cell numbers in astrocytes treated with LPS (1 *μ*g/ml) and the effect of cotreatment with QRC (10 *μ*M), ALA (10 *μ*M), or CRC (10 *μ*M) (b) and LYC (10 *μ*M), OLP (100 *μ*g/ml), NAC (300 *μ*M), or GTE (12.5 *μ*g/ml) (c). (d) Effect of the pool on TNF*α*- or LPS-induced proliferation of astrocytes. Data in (a–d) are the mean ± SEM of five independent experiments. (e) Effect of antioxidants at low doses used in the pool. (f) BrdU incorporation in cortical astrocytes stimulated with LPS (1 *μ*g/ml) for 7 days and the antiproliferative effect of the indicated antioxidants and the pool. Data, expressed as percent of CTR, are the mean ± SEM of three independent experiments, each performed in duplicate. ^∗^*p* ≤ 0.05 , ^∗∗^*p* ≤ 0.01, and ^∗∗∗^*p* ≤ 0.001 versus CTR (ANOVA and Dunnett's multiple comparison test).

Glial activation by inflammatory cytokines causes increased astrocytic proliferation [10] and formation of a glial scar that may limit neuronal repair [7, 9]. To assess the effect of antioxidant molecules on glial proliferation, we used cortical astrocytes activated by TNF*α* (10 ng/ml) or LPS (1 *μ*g/ml).  Figure 1 shows that the number of astrocytes dramatically increases (2-5-fold) during a 14-day time course (to simulate prolonged chronic glial activation) both in TNF*α*-treated astrocytes (Figures 1(a) and 1(d)) and, to a lesser extent, in LPS-treated cultures (Figures 1(b) and 1(c)). Both TNF*α*- and LPS-induced proliferations are prevented by cotreatment with RSV (10 *μ*M) by 4 or 7 days, respectively (Figure 1(a)).

LPS-induced astrocytic growth during a 7-day time course is also reduced by either QRC (10 *μ*M), ALA (10 *μ*M), or CRC (10 *μ*M) (Figure 1(b)), as well as by LYC (10 *μ*M), OLP (100 *μ*g/ml), NAC (300 *μ*M), or GTE (12.5 *μ*g/ml) (Figure 1(c)). Selection of antioxidant concentrations was based on their dose-dependent effect on astrocytic and neuronal survival, which were similar to the doses found to be effective on neuronal PC12 cells [28].

We previously reported that effective neuroprotection was achieved with defined cocktails of antioxidants [28]. Interestingly, we found that both TNF*α*- and LPS-induced proliferations are reduced in astrocytes cultured with a defined cocktail of selected antioxidant molecules at lower concentrations (pool = RSV 5 *μ*M, QRC 5 *μ*M, OLP 7 *μ*g/ml, and NAC 60 *μ*M) (Figure 1(d)). No effect was seen with the single antioxidants at the low doses used in the cocktail (Figure 1(e)).

Reduction of the cell number can be due to either decreased proliferation or decreased cell survival. The effect of antioxidants on astrocytic growth was further examined in the presence of BrdU for 24 h. We found that astrocyte treatment with LPS for 7 days promotes a 60% increase of BrdU incorporation (*p* ≤ 0.01) that is fully prevented by cotreatment with either RSV (10 *μ*M), QRC (10 *μ*M), ALA (10 *μ*M), CRC (10 *μ*M), LYC (10 *μ*M), GTE (12.5 *μ*g/ml), or NAC (300 *μ*M) or partially reduced by OLP (100 *μ*g/ml) (Figure 1(f)). Reduction of BrdU incorporation is also observed in astrocytes treated with LPS in the presence of the defined pool (Figure 1(f)). These data confirmed that the decrease of cell growth elicited by these molecules is due to inhibition of astrocytic proliferation and not the consequence of decreased survival.

### 3.2. Antioxidants Improve Astrocytic Viability during Inflammatory Stimuli and Oxidative Stress

**Figure 2:**
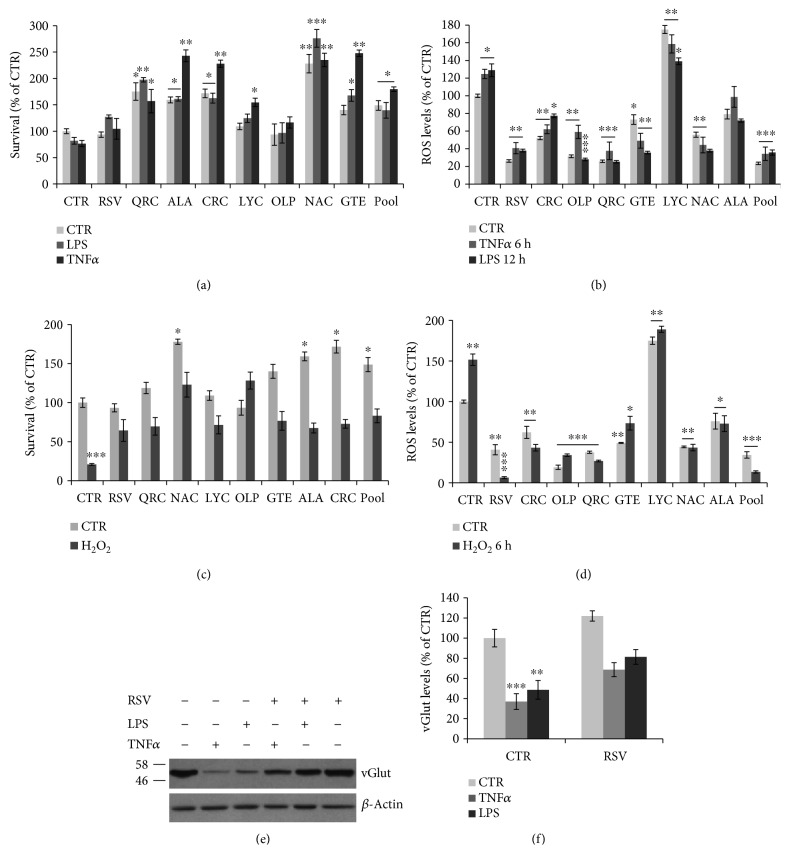
Effect of antioxidants on ROS levels and astrocyte viability. (a) Survival by the MTT assay of cortical astrocytes stimulated with TNF*α* (10 ng/ml) or LPS (1 *μ*g/ml) for 24 h and the effect of RSV (10 *μ*M), QRC (10 *μ*M), ALA (10 *μ*M), CRC (10 *μ*M), LYC (10 *μ*M), OLP (100 *μ*g/ml), GTE (12.5 *μ*g/ml), NAC (300 *μ*M), or pool. (b) Quantitation of astrocytic ROS levels by FACS analysis of DCH2F-DA fluorescence following stimulation with TNF*α* (10 ng/ml) for 6 h, or LPS (1 *μ*g/ml) for 12 h, and effect of the indicated antioxidants and pool. (c) MTT assay on astrocytes exposed to H_2_O_2_ (200 *μ*M) for 24 h in the presence or absence of the indicated antioxidants and pool. (d) ROS levels in astrocytes treated with H_2_O_2_ (200 *μ*M) for 6 h in the presence or absence of the indicated antioxidants and pool. All data are expressed as percent of CTR. MTT data are the mean ± SEM of three independent experiments, each performed with 4-6 samples for each treatment. ROS data are the mean ± SEM of three independent experiments, each performed in duplicate. (e, f) Western blot analysis and quantitation of vGLUT levels in astrocytes treated for 24 h with TNF*α* (10 ng/ml) or LPS (1 *μ*g/ml) and the effect of RSV cotreatment. Data, expressed as percent of CTR, are the mean ± SEM of three independent experiments. ^∗^*p* ≤ 0.05, ^∗∗^*p* ≤ 0.01, and ^∗∗∗^*p* ≤ 0.001 versus CTR (ANOVA and Dunnett's multiple comparison test).

Neuroinflammation is characterized by increased ROS production by activated microglia [11, 12]. Therefore, we examined whether antioxidants sustain astrocytic survival under oxidative stress, a condition linked to neuroinflammation. Indeed, we found that astrocyte viability is slightly decreased by TNF*α* (24%) or LPS (20%) for 24 h but is improved during cotreatment with RSV, LYC, or OLP or significantly enhanced by QRC, ALA, CRC, NAC, or GTE (*p* ≤ 0.05, 0.01, or 0.001), as compared to CTR or TNF*α*/LPS-treated samples (Figure 2(a)). The effect on survival was associated to a decrease of ROS levels. Time course studies showed that astrocytic ROS levels are not greatly changed by treatment with TNF*α* (10 ng/ml) or LPS (1 *μ*g/ml) for 6-12-24-48-72 h (data not shown); a modest but significant induction of intracellular ROS content is found in cultures treated with TNF*α* (23%) or LPS (29%) for 6 or 12 h, respectively (Figure 2(b)). Both basal and TNF*α*/LPS-induced ROS are significantly decreased by treatment with RSV, QRC, ALA, CRC, OLP, NAC, or GTE (Figure 2(b)). No effect was seen with LYC, but an interference with the ROS-sensitive dye is possible, as evident in Figure 2(d). All antioxidants are also able to deplete both basal and H_2_O_2_-induced intracellular ROS (Figure 2(d)) and partially or fully prevent the 80% reduction of survival induced by H_2_O_2_ (200 *μ*M) treatment for 12 h (Figure 2(c)). In all these conditions (both TNF*α*/LPS- and H_2_O_2_-treated astrocytes), ROS levels and cell viability are efficiently preserved by the antioxidant cocktail (Figures 2(a)–2(d)), suggesting that supplementation of low-dose antioxidant cocktail can protect astrocytes against mechanisms triggered by neuroinflammation and oxidative stress.

Alteration of astrocytic function following glial activation has been shown to reduce levels of vGLUT [10]. In agreement with previous studies, we found that both TNF*α* (10 ng/ml) and LPS (1 *μ*g/ml) treatments for 24 h significantly decrease vGLUT levels, which are partially restored by cotreatment with RSV (Figures 2(e) and 2(f)), suggesting that RSV is able to rescue proper astrocytic function.

### 3.3. Effect of Antioxidant Molecules on Neuroprotection against Reactive Gliosis-Induced Toxicity

**Figure 3:**
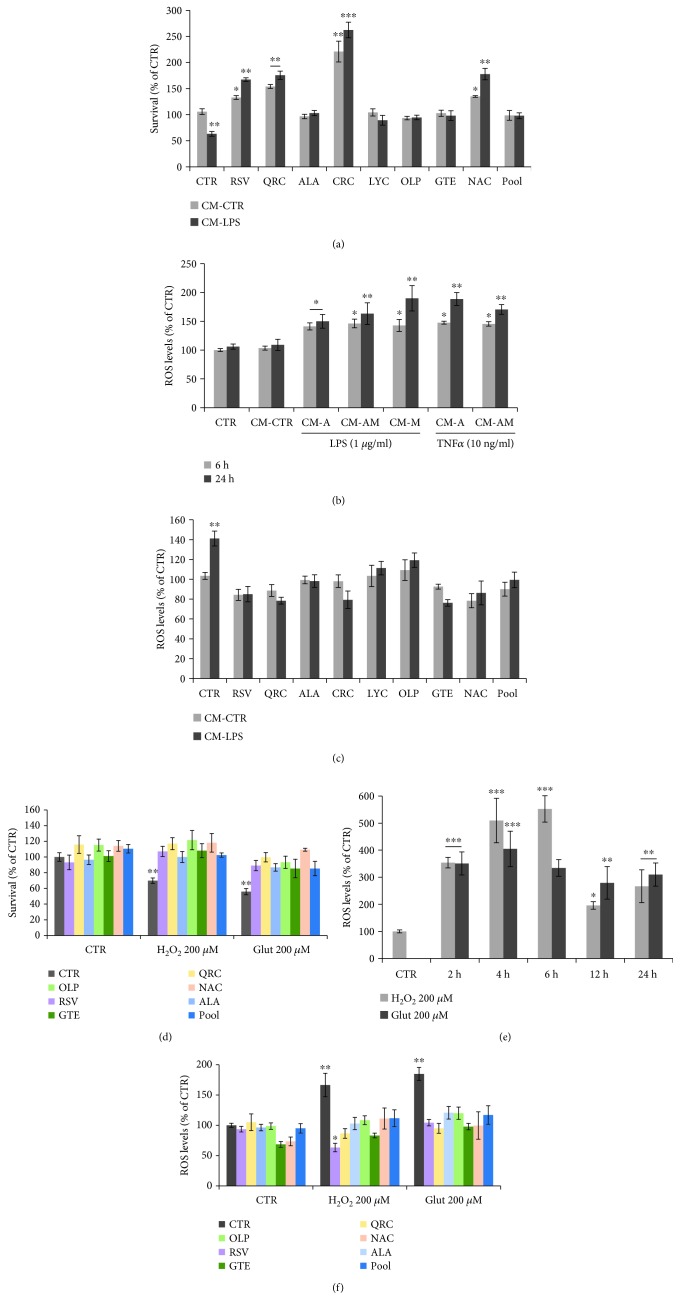
Effect of antioxidant molecules on neuronal ROS levels and survival. (a) MTT assay of cortical neurons exposed for 24 h to CM-LPS from LPS-stimulated astrocytes. Where indicated, neurons were preincubated ON with RSV (10 *μ*M), QRC (10 *μ*M), ALA (10 *μ*M), CRC (10 *μ*M), LYC (10 *μ*M), OLP (100 *μ*g/ml), GTE (12.5 *μ*g/ml), NAC (300 *μ*M), or pool-R. (b) Quantitation of neuronal ROS levels by FACS analysis of DCH2F-DA fluorescence following exposure to CM from LPS- or TNF*α*-stimulated astrocytes for 6 or 24 h. (c) Neuronal ROS levels following a 6 h treatment with CM-LPS. Where indicated, neurons were preincubated with the indicated antioxidants or the pool. (d) Survival of cortical neurons following 12 h treatment with H_2_O_2_ (200 *μ*M) or Glut (200 *μ*M), in the presence/absence of the indicated antioxidants or the lower concentration pool. (e) Time course of neuronal ROS production during incubation with H_2_O_2_ (200 *μ*M) or Glut (200 *μ*M). (f) Neuronal ROS levels following treatment with H_2_O_2_ (200 *μ*M) or Glut (200 *μ*M). Where indicated, neurons were preincubated with the indicated antioxidants or the pool. Data are expressed as percent of CTR. MTT data are the mean ± SEM of three independent experiments, each performed with 4-6 samples for each treatment. ROS data are the mean ± SEM of three experiments in duplicate. ^∗^*p* ≤ 0.05, ^∗∗^*p* ≤ 0.01, and ^∗∗∗^*p* ≤ 0.001 versus CTR (ANOVA and Dunnett's multiple comparison test).

Cytokines and chemokines released by activated glial cells during chronic neuroinflammation can compromise neuronal function [7, 9]. To assess glia-mediated neurotoxicity, cortical neurons were exposed for 24 h to conditioned medium (CM-LPS) prepared from astrocytes cultured in the presence of LPS (1 *μ*g/ml) for 48 h. Data in Figure 3(a) show that CM-LPS causes a 40% decrease of neuronal viability, as compared to cortical neurons treated with CM from untreated astroglial cells (CM-CTR). Loss of neuronal survival is fully prevented or significantly enhanced in cortical neurons cotreated with either RSV (10 *μ*M), QRC (10 *μ*M), ALA (10 *μ*M), CRC (10 *μ*M), LYC (10 *μ*M), OLP (100 *μ*g/ml), GTE (12.5 *μ*M), or NAC (300 *μ*M) before addition of CM-LPS (Figure 3(a)). The effect of antioxidants on neuronal survival was also evident after treatment of neurons with CM-TNF*α* (data not shown) and associated with decreased intracellular ROS content. In fact, ROS production shows a 50-80% rise in neurons exposed for 6 or 24 h to CM-LPS or CM-TNF*α* from astrocytes (CM-A), or microglia (CM-M), or mixed astroglial cells (CM-AM), as compared to CM-CTR (Figure 3(b)), suggesting that neuronal oxidative stress can be ascribed to both activated astrocytes and microglia. ROS production induced by CM-LPS for 6 h is restored to basal levels by RSV, QRC, ALA, CRC, GTE, or NAC or slightly lowered by LYC or OLP (Figure 3(c)). It is remarkable that neuroprotection against CM-LPS-mediated toxicity is also achieved with the cocktail (antioxidant pool) (Figures 3(a) and 3(c)).

Glutamate excitotoxicity is known to be part of the oxidative stress response following glial activation [7, 9]. Therefore, we examined the effect of antioxidant molecules in neuroprotection against oxidative stress and glutamate toxicity. As shown in Figure 3(d), treatment for 12 h with H_2_O_2_ (200 *μ*M) or Glut (200 *μ*M) causes a 30% and 45% decrease of neuronal viability, respectively. Time course studies showed that both stimuli also cause a 3-6-fold increase of ROS levels (Figure 3(e)). Both survival and ROS content are restored in neurons cotreated with RSV, QRC, ALA, OLP, GTE, or NAC, as well as by the antioxidant cocktail (Figures 3(d) and 3(f)). These data indicate that the low-dose cocktail of antioxidants is effective in neuroprotection against these two components of neuroinflammation.

### 3.4. Differential Regulation of NF-*κ*B in Neurons and Astrocytes under Conditions of Reactive Gliosis

Modulation of cell survival and function involves modulation of gene transcription. Among transcription factors, we focused on NF-*κ*B whose binding activity is modulated by survival signaling molecules and inflammatory responses [17–19].

**Figure 4:**
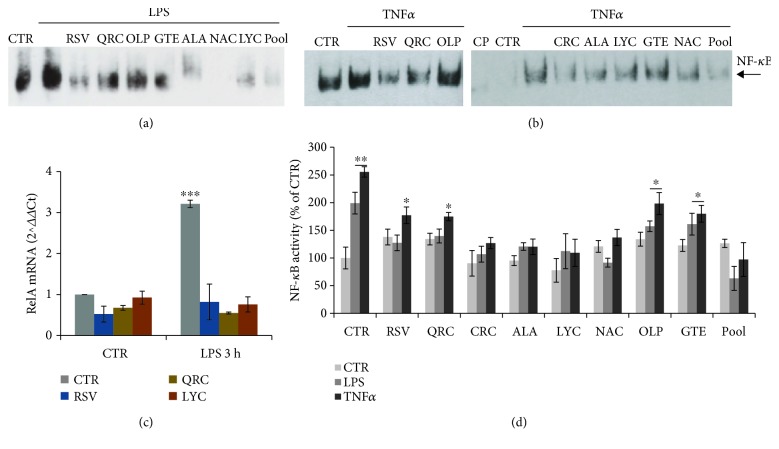
Effect of antioxidants on astrocytic NF-*κ*B binding activity. (a, b) EMSA of nuclear extracts from astrocytes stimulated for 2 h with LPS (a) or TNF*α* (b) and the effect of RSV (10 *μ*M), QRC (10 *μ*M), OLP (100 *μ*g/ml), GTE (12.5 *μ*g/ml), CRC (10 *μ*M), ALA (10 *μ*M), LYC (10 *μ*M), NAC (300 *μ*M), or pool. CP = competition assay with excess unlabeled oligonucleotide. (c) qRT-PCR on total RNA prepared from astrocytes treated with LPS for 3 h. Where indicated, astrocytes were preincubated with RSV (10 *μ*M), QRC (10 *μ*M), or LYC (10 *μ*M). Data, expressed as fold of induction (*ΔΔ*Ct), are the mean of two independent experiments. (d) Quantitative analysis of NF-*κ*B binding activity by ImageJ software. Data, expressed as percent of CTR, are the mean ± SEM of three experiments. ^∗^*p* ≤ 0.05, ^∗∗^*p* ≤ 0.01, and ^∗∗∗^*p* ≤ 0.001 versus CTR (ANOVA and Dunnett's multiple comparison test).

We first assessed the effect of LPS treatment on astrocytes by RT-PCR analysis of p65/RelA, the main component of NF-*κ*B. We found that exposure of astrocytes to LPS (1 *μ*g/ml) for 3 h causes a 3-fold induction of p65/RelA mRNA content that is strongly prevented in astrocytes pretreated for 30 min with RSV (10 *μ*M), QRC (10 *μ*M), or LYC (10 *μ*M) before addition of LPS (Figure 4(c)). A slight reduction of p65/RelA mRNA levels by RSV, but not by QRC or LYC, is also observed at 6 h (data not shown).

In addition, we investigated NF-*κ*B activity by EMSA. We found that NF-*κ*B binding activity is strongly induced in astrocytes treated with LPS (1 *μ*g/ml) for 2 h (Figures 4(a) and 4(d)). The intensity of DNA-protein complexes is lowered in nuclear extracts isolated from astrocytes preincubated for 30 min with RSV (10 *μ*M), QRC (10 *μ*M), CRC (10 *μ*M), ALA (10 *μ*M), LYC (10 *μ*M), or NAC (300 *μ*M). LPS-induced NF-*κ*B activity is not significantly affected by OLP (100 *μ*g/ml) or GTE (12.5 *μ*g/ml) (Figures 4(a) and 4(d)). The inhibitory activity of these molecules on NF-*κ*B binding is also observed during treatments with TNF*α* (10 ng/ml) (Figures 4(b) and 4(d)). Because of the role of NF-*κ*B in regulating proinflammatory genes (such as cytokines), our data suggest that antioxidant molecules might efficiently reduce deleterious effects of the glial inflammatory loop due to excess cytokine release.

**Figure 5:**
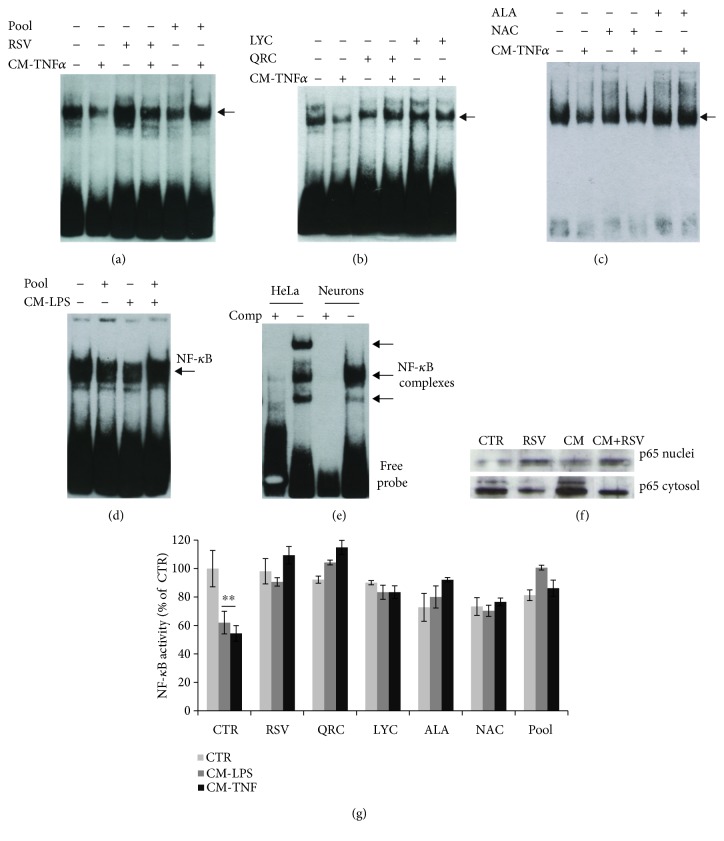
Effect of antioxidants on neuronal NF-*κ*B binding activity. (a–d) EMSA of nuclear extracts prepared from cortical neurons treated for 2 h with CM from astrocytes stimulated with TNF*α* (a–c) or LPS (d). Where indicated, neurons were pretreated for 30 min with RSV (10 *μ*M) (a), QRC (10 *μ*M) or LYC (10 *μ*M) (b), ALA (10 *μ*M) or NAC (300 *μ*M) (c), or the pool (a, d). (e) Competition assay with excess unlabeled oligonucleotide on nuclear extracts from neurons treated with TNF*α* or HeLa cells (positive CTR, EMSA NF-*κ*B kit, Panomics). (f) Western blot analysis of p65 protein in cytosol or nuclear extracts from neurons treated with CM-LPS. (g) Quantitative analysis of NF-*κ*B binding activity by ImageJ software. Data, expressed as percent of CTR, are the mean ± SEM of three experiments. ^∗∗^*p* ≤ 0.01 versus CTR (ANOVA and Dunnett's multiple comparison test).

NF-*κ*B plays also an important role in neuronal survival. It was remarkable to observe an opposite trend of NF-*κ*B binding activity in cortical neurons. EMSA of nuclear extracts revealed that CM-TNF*α* (Figures 5(a)–5(c) and 5(g)) or CM-LPS (Figures 5(d) and 5(g)) cause a significant reduction of NF-*κ*B binding activity that is prevented by pretreatment for 30 min with RSV (10 *μ*M) (Figures 5(a) and 5(g)), QRC (10 *μ*M) or LYC (10 *μ*M) (Figures 5(b) and 5(g)), and ALA (10 *μ*M) or NAC (10 *μ*g/ml) (Figures 5(c) and 5(g)). The binding was specific, as it was fully competed by addition of excess unlabeled oligonucleotide (Figures 4(b) and 5(e)). The effect of RSV on NF-*κ*B was further confirmed by Western blot analysis showing cytosolic p65 protein accumulation in neurons treated with CM-LPS (Figure 5(f)). It is remarkable that NF-*κ*B binding activity is restored by the defined antioxidant pool both in cortical astrocytes (Figures 4(a), 4(b), and 4(d)) and in cortical neurons (Figures 5(a), 5(d), and 5(g)).

### 3.5. Effect of Glial Activation and RSV on Neuronal Transcription Factors

**Figure 6:**
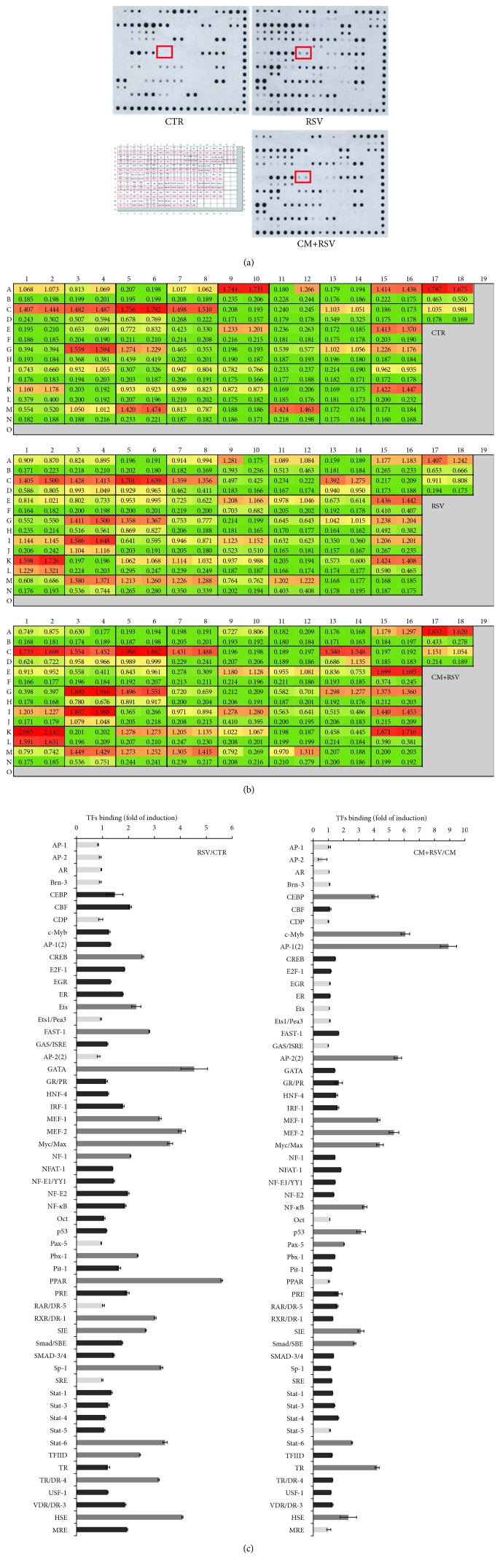
Modulation of neuronal transcription factor activity by glial activation and RSV. (a) Protein-DNA array of nuclear extracts prepared from cortical neurons treated for 2 h with CM (from LPS-stimulated astrocytes), RSV (10 *μ*M), or CM-LPS plus RSV. The grid for location of transcription factors (https://www.biocat.com/bc/pdf/MA1210_MA1215_Protein_DNA_Arrays). (b) Quantitation of transcription factors. (c) Bar graph for quantitative analysis of the ratio RSV/CTR and CM-LPS+RSV/CM-LPS. Light grey square = no change or reduction versus CTR; dark grey square = induction versus CTR (1 > 2‐fold); grey square = induction versus CTR (>2‐fold).

To investigate the effect of glial activation on neuronal gene transcription, we analyzed DNA binding activity of various transcription factors by protein-DNA array. Consistent with the EMSA data, protein-DNA arrays revealed a decrease of the NF-*κ*B signal in nuclear extracts prepared from neurons treated for 2 h with CM-LPS and a significant induction (about 2-fold) in RSV and in CM+RSV-treated neurons (Figures 6(a)–6(c)), thus confirming the role of decreased NF-*κ*B binding activity in glia-mediated toxicity of neurons. In addition, quantitation of transcription factors showed that neuronal response to RSV involves a marked increase of binding activity for several transcription factors (CEBP, c-Myb, AP-1(2), AP-2(2), MEF-1/2, Myc/Max, SIE, Smad/SBE, Stat-6, TR, and HSE) (Figure 6(c)), suggesting that RSV has a crucial role in modulating neuronal gene expression. The role of these proteins in RSV-mediated action is currently under investigation.

## 4. Discussion

Epidemiological studies indicate that diets based on vegetables, fruit, and fish consumption are healthy and protect from cancer and neurodegenerative diseases. Healthy dietary factors (i.e., polyphenols, carotenoids, and thiolic compounds) are known for their antioxidant activity. In addition, they have a marked anti-inflammatory action and influence cell metabolism by modulating the activity of enzymes, nuclear receptors, and transcription factors [35, 36].

Neuroinflammation and oxidative stress underlie neuronal and astrocytic dysfunction in neurodegenerative diseases [3, 11, 12]. In this study, we demonstrate that distinct classes of dietary antioxidants (RSV, QRC, ALA, CRC, LYC, OLP, NAC, and GTE) are able to (i) rescue neuronal viability (Figure 3) and (ii) astrocytic function (Figure 2) through mechanisms that involve (iii) reduction of astrocyte proliferation (Figure 1) and (iv) decrease of neuronal and astrocytic ROS production (Figures 2 and 3).

Our data are in agreement with a huge number of studies showing the beneficial antioxidant activity and neuroprotection of these molecules on neuronal and glial cells, both in vitro and animal models of neurodegeneration, such as *β*-amyloid, or MPTP, or glutamate toxicity [22–28, 30–34]. In this regard, it is remarkable that RVS upregulates levels of the glutamate transporter vGLUT, in agreement with other studies showing that RSV improves astrocytic function by increasing glutamate uptake and glutamine synthetase activity [40–42]. Similar changes were found for ALA [43].

Antioxidants have been found to act through activation of a variety of signaling pathways. We focused on NF-*κ*B, a multifunctional transcription factor regulating survival and proinflammatory genes in response to a variety of stress conditions that affect cellular homeostasis. A number of studies have shown that NF-*κ*B is modulated by antioxidant molecules [27, 31, 44, 45]. Interestingly, we found that NF-*κ*B binding activity changed in a cell-specific manner. Specifically, NF-*κ*B binding activity increased in astrocytes treated with TNF*α* or LPS, sustaining current knowledge about the critical role of NF-*κ*B in the glial inflammatory loop fostering cytokine production and neurotoxicity. In neurons, constitutive NF-*κ*B activity is suggested to connect neuronal activity to cell survival pathways [46, 47]. Accordingly, we found that NF-*κ*B binding activity was reduced by about 40-50% in neurons exposed to the “proinflammatory medium” (CM) from activated astrocytes. Remarkably, all tested molecules fully or partially restored basal conditions. These data are in line with our dynamic model of ROS management based on mathematical systems biology modeling, which shows the complex molecular network connecting NF-*κ*B to Nrf2/Keap1/ARE in response to oxidative stress and how this pathway is regulated by a large number of stress sensors (DJ-1, Parkin, etc.) that act differently in different cellular contexts and perturbations to regulate mitochondrial function (Colangelo-Alberghina-Papa, unpublished).

Finally, it was remarkable that the effect of antioxidants on astrocytic proliferation, neuronal survival, and intracellular ROS levels was efficiently achieved by treating cells with a defined cocktail of selected antioxidants at low concentrations (pool = RSV 5 *μ*M, QRC 5 *μ*M, OLP 7 *μ*g/ml, and NAC 60 *μ*M) (Figures 1–3). These data suggest that efficient neuronal and astrocytic functions are sustained by combinations of molecules belonging to distinct groups of dietary antioxidants (flavonoids, nonflavonoids, carotenoids, and thiolic compounds) at concentrations lower than those required for efficacy of each single molecule. These results are in line with our previous data of antioxidant efficacy within a strict range of concentrations in the low *μ*M range. For instance, RSV displays neurotrophic properties on cortical neurons and neuronal PC12 [28] at very low concentrations (1-10 *μ*M), while higher concentrations were toxic, in agreement with its well-known hormesis effect [28, 29]. Defined antioxidant cocktails were found to promote effective neuroprotection on NGF-deprived neuronal cells by depleting ROS levels and improving mitochondrial function [28].

In natural food, antioxidants are usually present in limited quantities and absorbed at even lower amounts. Moreover, it is well known that antioxidants have pleiotropic effects [35]. For instance, some dietary antioxidants including polyphenols can downregulate the production of proinflammatory mediators, while other molecules can promote several biological functions in resting cells. Therefore, it is likely that administration of multiple dietary factors at low doses can mimic physiological conditions. Accordingly, the combined effects of two or more antioxidants have been reported [23, 28, 48–51]. However, synergistic protection by low-dose antioxidant cocktails has not been described so far in primary cultures of neurons and astrocytes. It was conceivable that the efficacy of our cocktail could be ascribed to the complementary biological activity of dietary antioxidants of the pool in modulating metabolism and mitochondrial function.

In conclusion, our study (i) provides evidence about the role of distinct dietary supplements in promoting neuronal and astrocytic function through cell-specific modulation of NF-*κ*B and (ii) newly identifies a defined low-dose “physiological” antioxidant cocktail that reduces mechanisms of reactive gliosis and promotes neuroprotection.

## Data Availability

Data used to support the findings of this study are included within the article.

## Abbreviations

BrdU: Bromodeoxyuridine
DCH2F-DA: 2′,7′-Dichlorodihydrofluorescein diacetate
EMSA: Electrophoretic mobility shift assay
NF-*κ*B: Nuclear factor kappa-light-chain-enhancer of activated B cells
ROS: Reactive oxygen species
ALA: Alpha-lipoic acid
CRC: Curcumin
GTE: Green tea extract
LYC: Lycopene
NAC: N-Acetyl cysteine
OLP: Oliplus
QRC: Quercetin
RSV: Resveratrol.

## References

[1] Mancuso C., Scapagini G., Currò D. (2007). Mitochondrial dysfunction, free radical generation and cellular stress response in neurodegenerative disorders. *Frontiers in Bioscience*.

[2] Lassmann H. (2014). Multiple sclerosis: Lessons from molecular neuropathology. *Experimental Neurology*.

[3] Fischer R., Maier O. (2015). Interrelation of oxidative stress and inflammation in neurodegenerative disease: role of TNF. *Oxidative Medicine and Cellular Longevity*.

[4] Leszek J., Barreto G. E., Gąsiorowski K., Koutsouraki E., Ávila-Rodrigues M., Aliev G. (2016). Inflammatory mechanisms and oxidative stress as key factors responsible for progression of neurodegeneration: role of brain innate immune system. *CNS & Neurological Disorders-Drug Targets*.

[5] Adamczyk B., Adamczyk-Sowa M. (2016). New insights into the role of oxidative stress mechanisms in the pathophysiology and treatment of multiple sclerosis. *Oxidative Medicine and Cellular Longevity*.

[6] Rodríguez-Arellano J. J., Parpura V., Zorec R., Verkhratsky A. (2016). Astrocytes in physiological aging and Alzheimer’s disease. *Neuroscience*.

[7] Colangelo A. M., Alberghina L., Papa M. (2014). Astrogliosis as a therapeutic target for neurodegenerative diseases. *Neuroscience Letters*.

[8] Montana V., Malarkey E. B., Verderio C., Matteoli M., Parpura V. (2006). Vesicular transmitter release from astrocytes. *Glia*.

[9] Sofroniew M. V. (2009). Molecular dissection of reactive astrogliosis and glial scar formation. *Trends in Neurosciences*.

[10] Cirillo G., Colangelo A. M., Berbenni M. (2015). Purinergic modulation of spinal neuroglial maladaptive plasticity following peripheral nerve injury. *Molecular Neurobiology*.

[11] Gao H. M., Zhou H., Hong J. S. (2012). NADPH oxidases: novel therapeutic targets for neurodegenerative diseases. *Trends in Pharmacological Sciences*.

[12] Qin L., Liu Y., Hong J. S., Crews F. T. (2013). NADPH oxidase and aging drive microglial activation, oxidative stress, and dopaminergic neurodegeneration following systemic LPS administration. *Glia*.

[13] Ramassamy C. (2006). Emerging role of polyphenolic compounds in the treatment of neurodegenerative diseases: a review of their intracellular targets. *European Journal of Pharmacology*.

[14] Gan L., Mucke L. (2008). Paths of convergence: sirtuins in aging and neurodegeneration. *Neuron*.

[15] Albarracin S. L., Stab B., Casas Z. (2012). Effects of natural antioxidants in neurodegenerative disease. *Nutritional Neuroscience*.

[16] Rehman M. U., Wali A. F., Ahmad A. (2019). Neuroprotective strategies for neurological disorders by natural products: an update. *Current Neuropharmacology*.

[17] Jha N. K., Jha S. K., Kar R., Nand P., Swati K., Goswami V. K. (2019). Nuclear factor-kappa *β* as a therapeutic target for Alzheimer’s disease. *Journal of Neurochemistry*.

[18] Li Q., Verma I. M. (2002). NF-*κ*B regulation in the immune system. *Nature Reviews Immunology*.

[19] Pugazhenthi S., Zhang Y., Bouchard R., Mahaffey G. (2013). Induction of an inflammatory loop by interleukin-1*β* and tumor necrosis factor-*α* involves NF-kB and STAT-1 in differentiated human neuroprogenitor cells. *PLoS One*.

[20] Bravo L. (1998). Polyphenols: chemistry, dietary sources, metabolism, and nutritional significance. *Nutrition Reviews*.

[21] Manach C., Williamson G., Morand C., Scalbert A., Rémésy C. (2005). Bioavailability and bioefficacy of polyphenols in humans. I. Review of 97 bioavailability studies. *The American Journal of Clinical Nutrition*.

[22] Jazvinšćak J. M., Vuković L., Puhović J., Erhardt J., Oršolić N. (2012). Neuroprotective effect of quercetin against hydrogen peroxide-induced oxidative injury in P19 neurons. *Journal of Molecular Neuroscience*.

[23] Bournival J., Quessy P., Martinoli M. G. (2009). Protective effects of resveratrol and quercetin against MPP+ -induced oxidative stress act by modulating markers of apoptotic death in dopaminergic neurons. *Cellular and Molecular Neurobiology*.

[24] Wight R. D., Tull C. A., Deel M. W. (2012). Resveratrol effects on astrocyte function: relevance to neurodegenerative diseases. *Biochemical and Biophysical Research Communications*.

[25] Lee J., Jo D. G., Park D., Chung H. Y., Mattson M. P. (2014). Adaptive cellular stress pathways as therapeutic targets of dietary phytochemicals: focus on the nervous system. *Pharmacological Reviews*.

[26] Currò M., Trovato-Salinaro A., Gugliandolo A. (2015). Resveratrol protects against homocysteine-induced cell damage via cell stress response in neuroblastoma cells. *Journal of Neuroscience Research*.

[27] Bellaver B., Bobermin L. D., Souza D. G. (2016). Signaling mechanisms underlying the glioprotective effects of resveratrol against mitochondrial dysfunction. *Biochimica et Biophysica Acta (BBA) - Molecular Basis of Disease*.

[28] Amara F., Berbenni M., Fragni M. (2015). Neuroprotection by cocktails of dietary antioxidants under conditions of nerve growth factor deprivation. *Oxidative Medicine and Cellular Longevity*.

[29] Calabrese E. J., Mattson M. P., Calabrese V. (2010). Resveratrol commonly displays hormesis: occurrence and biomedical significance. *Human & Experimental Toxicology*.

[30] Rao A. V., Rao L. G. (2007). Carotenoids and human health. *Pharmacological Research*.

[31] Hwang S., Lim J. W., Kim H. (2017). Inhibitory effect of lycopene on amyloid-*β*-induced apoptosis in neuronal cells. *Nutrients*.

[32] Salinthone S., Yadav V., Schillace R. V., Bourdette D. N., Carr D. W. (2010). Lipoic acid attenuates inflammation via cAMP and protein kinase A signaling. *PLoS One*.

[33] Bavarsad Shahripour R., Harrigan M. R., Alexandrov A. V. (2014). N-Acetylcysteine (NAC) in neurological disorders: mechanisms of action and therapeutic opportunities. *Brain and Behavior: A Cognitive Neuroscience Perspective*.

[34] Fiedler S. E., Yadav V., Kerns A. R. (2018). Lipoic acid stimulates cAMP production in healthy control and secondary progressive MS subjects. *Molecular Neurobiology*.

[35] Riccio P. (2011). The molecular basis of nutritional intervention in multiple sclerosis: a narrative review. *Complementary Therapies in Medicine*.

[36] Riccio P., Rossano R., Liuzzi G. M. (2010). May diet and dietary supplements improve the wellness of multiple sclerosis patients? A molecular approach. *Autoimmune Diseases*.

[37] Sala G., Marinig D., Riva C. (2016). Rotenone down-regulates HSPA8/hsc70 chaperone protein *in vitro*: a new possible toxic mechanism contributing to Parkinson’s disease. *Neurotoxicology*.

[38] Martorana F., Gaglio D., Bianco M. R. (2018). Differentiation by nerve growth factor (NGF) involves mechanisms of crosstalk between energy homeostasis and mitochondrial remodeling. *Cell Death & Disease*.

[39] Colangelo A. M., Johnson P. F., Mocchetti I. (1998). *β*-Adrenergic receptor-induced activation of nerve growth factor gene transcription in rat cerebral cortex involves CCAAT/enhancer-binding protein *δ*. *Proceedings of the National Academy of Sciences of the United States of America*.

[40] dos Santos A. Q., Nardin P., Funchal C. (2006). Resveratrol increases glutamate uptake and glutamine synthetase activity in C6 glioma cells. *Archives of Biochemistry and Biophysics*.

[41] de Almeida L. M., Piñeiro C. C., Leite M. C. (2007). Resveratrol increases glutamate uptake, glutathione content, and S100B secretion in cortical astrocyte cultures. *Cellular and Molecular Neurobiology*.

[42] Zhou H., Chen Q., Kong D. L., Guo J., Wang Q., Yu S. Y. (2011). Effect of resveratrol on gliotransmitter levels and p38 activities in cultured astrocytes. *Neurochemical Research*.

[43] Kleinkauf-Rocha J., Bobermin L. D., Machado Pde M., Gonçalves C. A., Gottfried C., Quincozes-Santos A. (2013). Lipoic acid increases glutamate uptake, glutamine synthetase activity and glutathione content in C6 astrocyte cell line. *International Journal of Developmental Neuroscience*.

[44] Pahan K., Sheikh F. G., Namboodiri A. M., Singh I. (1998). *N*-Acetyl cysteine inhibits induction of no production by endotoxin or cytokine stimulated rat peritoneal macrophages, C_6_ glial cells and astrocytes. *Free Radical Biology and Medicine*.

[45] Chen J., Zhou Y., Mueller-Steiner S. (2005). SIRT1 protects against microglia-dependent amyloid-*β* toxicity through inhibiting NF-*κ*B signaling. *Journal of Biological Chemistry*.

[46] Bhakar A. L., Tannis L. L., Zeindler C. (2002). Constitutive nuclear factor-*κ*B activity is required for central neuron survival. *The Journal of Neuroscience*.

[47] Gustin J. A., Ozes O. N., Akca H. (2004). Cell type-specific expression of the I*κ*B kinases determines the significance of phosphatidylinositol 3-kinase/Akt signaling to NF-*κ*B activation. *Journal of Biological Chemistry*.

[48] Conte A., Pellegrini S., Tagliazucchi D. (2003). Synergistic protection of PC12 cells from *β*-amyloid toxicity by resveratrol and catechin. *Brain Research Bulletin*.

[49] Grasso S., Bramanti V., Tomassoni D. (2014). Effect of lipoic acid and *α*-glyceryl-phosphoryl-choline on astroglial cell proliferation and differentiation in primary culture. *Journal of Neuroscience Research*.

[50] Aliev G., Liu J., Shenk J. C. (2009). Neuronal mitochondrial amelioration by feeding acetyl-L-carnitine and lipoic acid to aged rats. *Journal of Cellular and Molecular Medicine*.

[51] Zamin L. L., Filippi-Chiela E. C., Dillenburg-Pilla P., Horn F., Salbego C., Lenz G. (2009). Resveratrol and quercetin cooperate to induce senescence-like growth arrest in C6 rat glioma cells. *Cancer Science*.

